# Supplementary material for: What Is the Nutritional Quality of Pre-Packed Foods Marketed to Children in Food Stores? A Survey in Switzerland
Source: Nutrients. 2024 May 28;16(11):1656. doi: 10.3390/nu16111656 (PMC11175003; doi:10.3390/nu16111656)
Supplement: Supplementary file 1 [file nutrients-16-01656-s001.zip › nutrients-2997384-supplementary.pdf]

Supplementary Materials

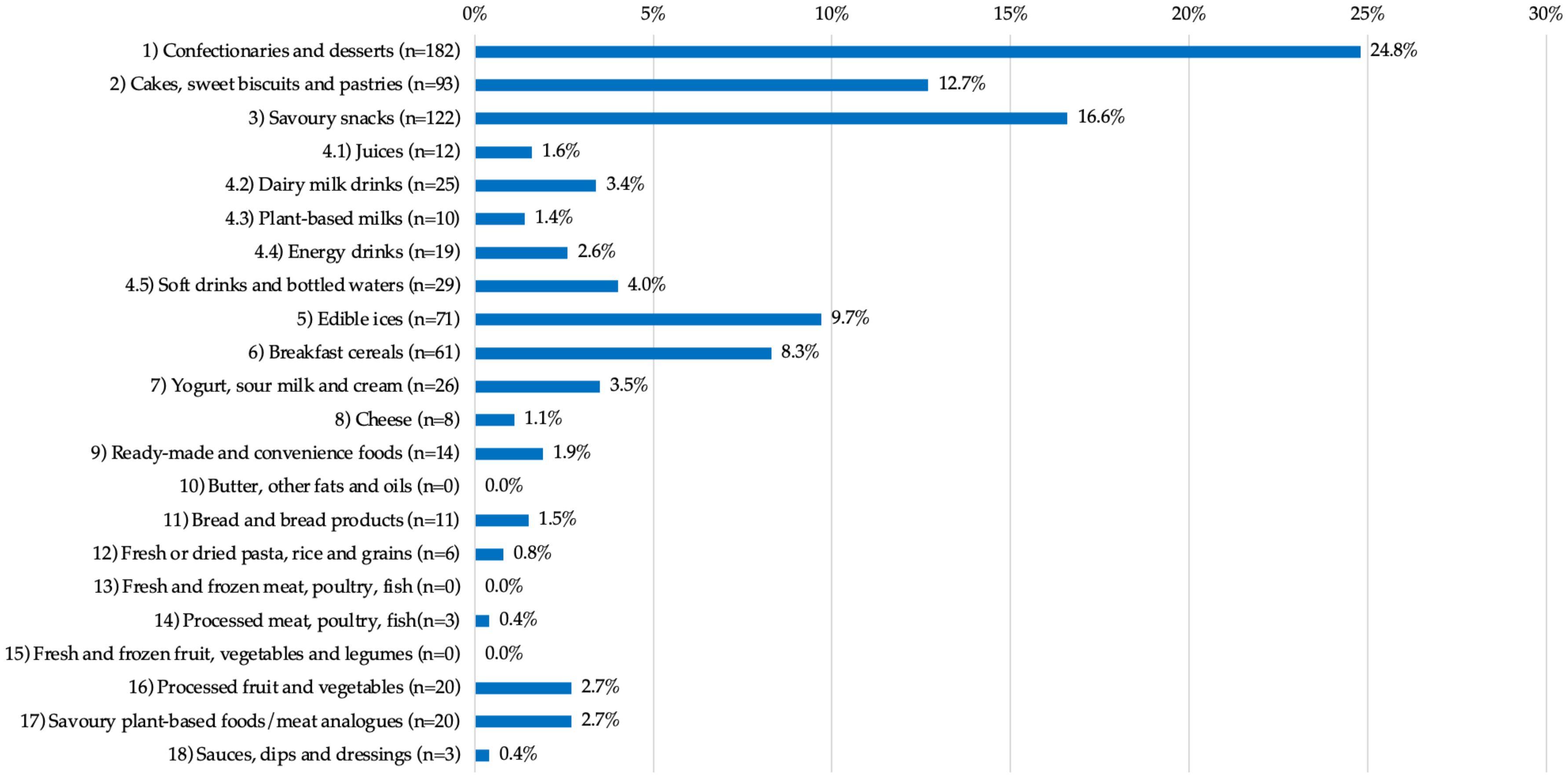

Figure S1: Proportions of food and beverages marketed to children by WHO NPM categories.

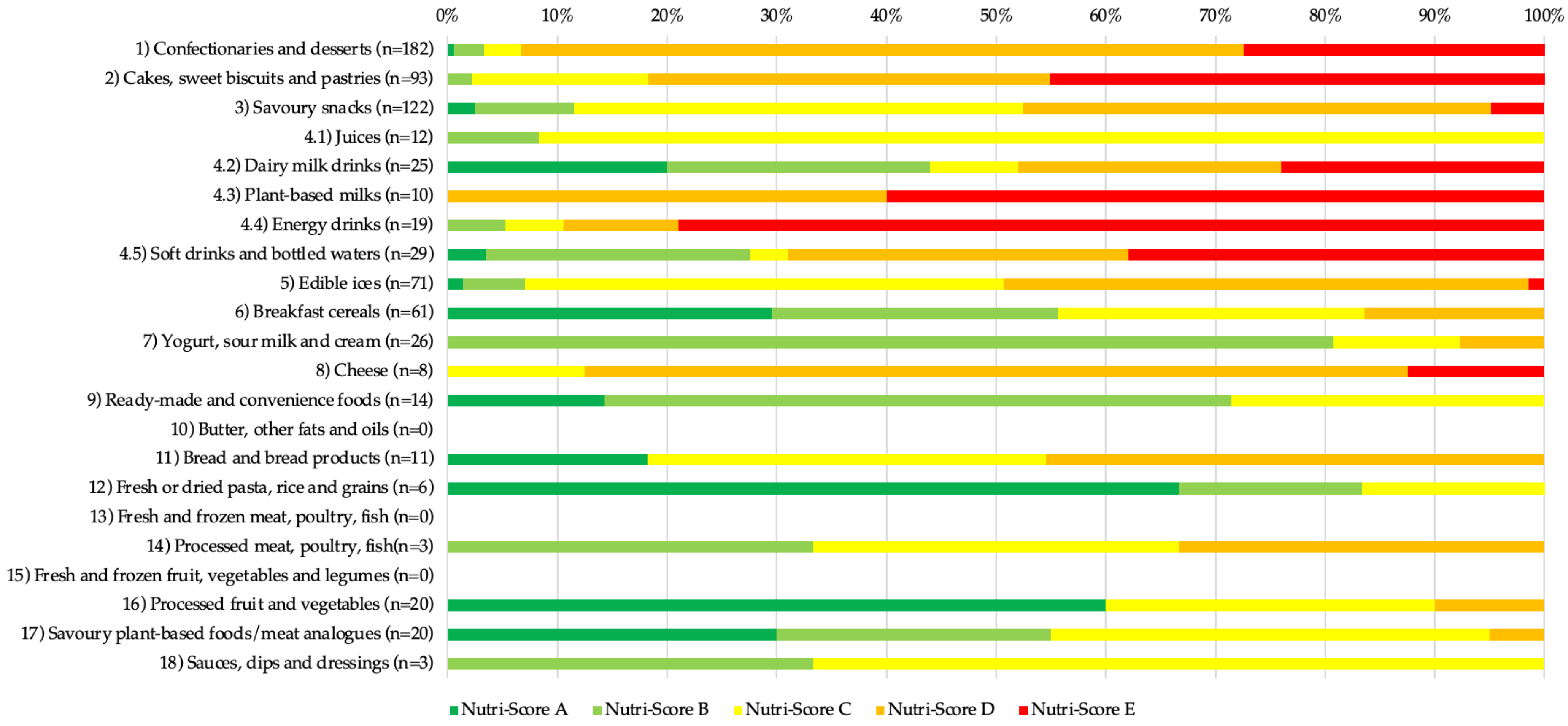

Figure S2: Proportions of Nutri-Score categories by WHO NPM categories in food and beverages marketed to children.

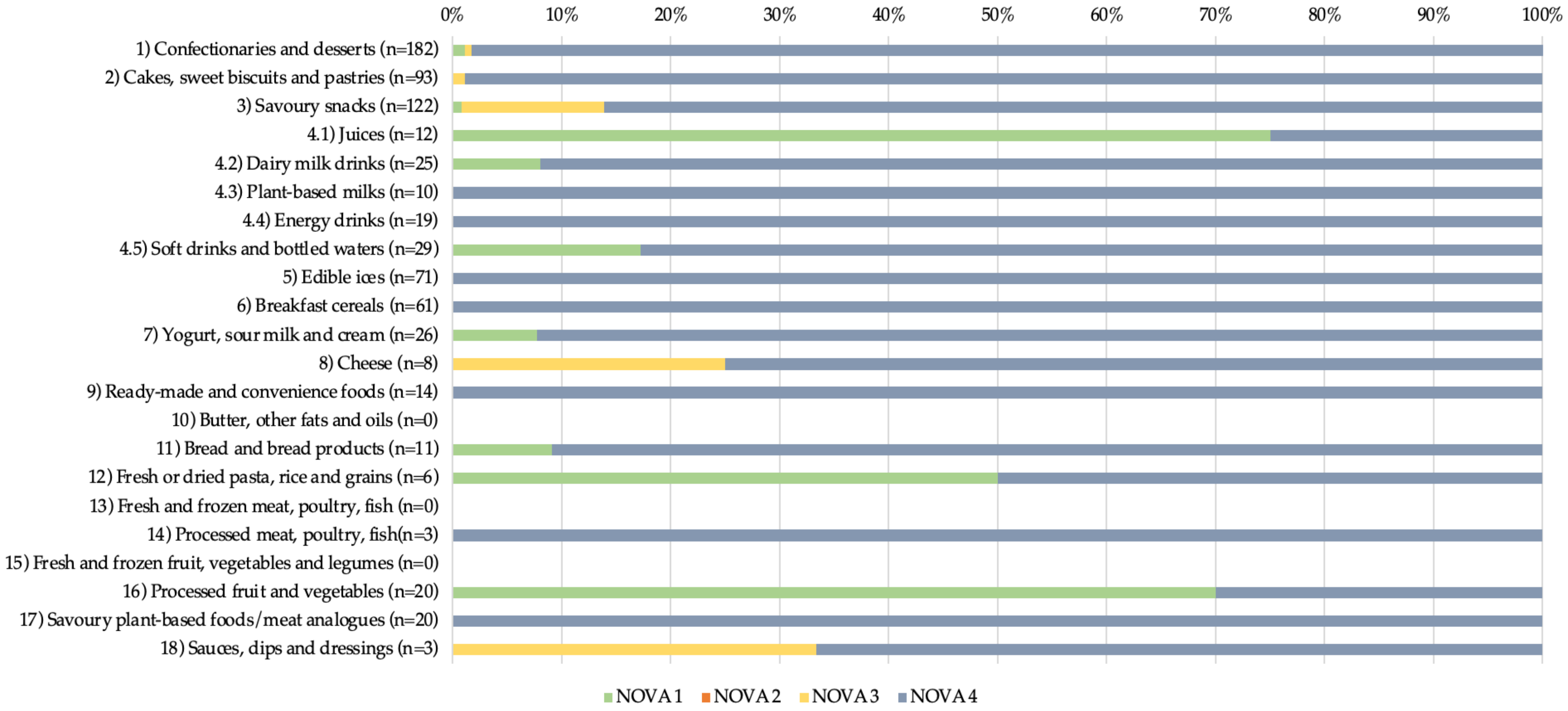

Figure S3: Proportions of NOVA categories by WHO NPM categories in food and beverages marketed to children.

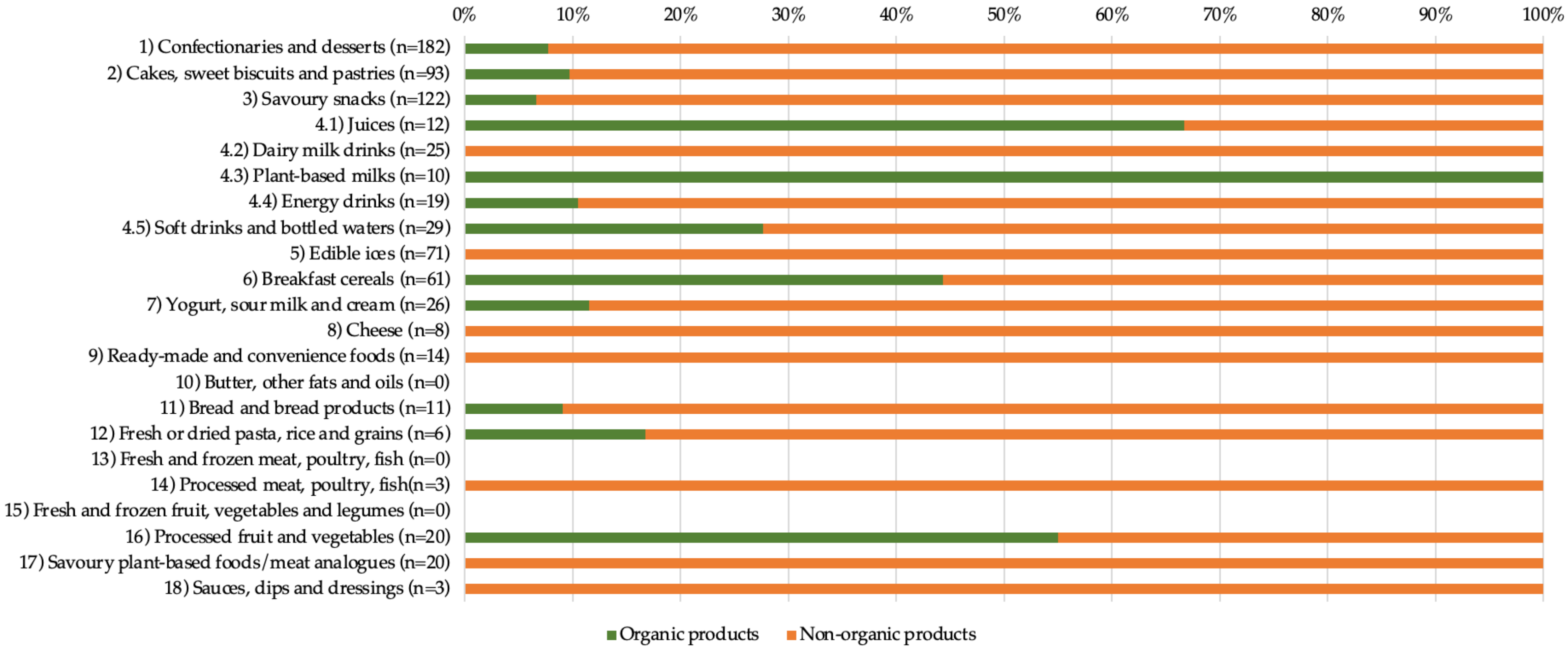

Figure S4: Proportions of WHO NPM categories by organic and non-organic categories in food and beverages marketed to children.
